# Real-world clinical effectiveness and exploratory longitudinal immunological changes of Efgartigimod in myasthenia gravis: a retrospective study and a single-cell analysis in one patient

**DOI:** 10.3389/fneur.2026.1980412

**Published:** 2026-10-19

**Authors:** Liting Tian, Lanxin Zhang, Jingnan Jin, Xia Wang, Zihong Chen, Lukuan Qiao, Qingling Guo, Yan Li, Jiayi Zhang, Yufan Zhao, Lihua Wang, Lei Li, Jianjian Wang

**Affiliations:** Department of Neurology, The Second Affiliated Hospital of Harbin Medical University, Harbin, China

**Keywords:** generalized myasthenia gravis, Efgartigimod, real-world study, single-cell sequencing, immunological changes

## Abstract

**Introduction:**

Efgartigimod, an antagonist of the neonatal Fc receptor, has demonstrated efficacy in generalized myasthenia gravis, but evidence from routine clinical practice remains limited, and its immunological effects beyond immunoglobulin G reduction are incompletely understood.

**Methods:**

We retrospectively evaluated the 12-week effectiveness and safety of Efgartigimod in 21 patients with acetylcholine receptor antibody-positive generalized myasthenia gravis. Outcomes included changes in Myasthenia Gravis Activities of Daily Living scores, response rates defined by reductions of ≥2 and ≥5 points, achievement of minimal symptom expression, changes in prednisone dosage, and adverse events. To explore potential longer-term immune changes, paired peripheral blood mononuclear cell samples obtained from one patient at baseline and after 6 months of repeated treatment were analyzed using single-cell RNA sequencing and T- and B-cell receptor repertoire sequencing.

**Results:**

The median MG-ADL score decreased from 7.0 (IQR, 6.0–8.0) at baseline to 1.0 (IQR, 1.0–1.5) at week 12. After the first and second treatment cycles, 85.7 and 94.7% of patients achieved CMI, respectively, while 57.1 and 84.2% achieved ≥5-point reductions. At week 12, 73.7% of patients achieved MSE. Treatment was generally well tolerated, with two mild adverse events and no serious adverse events. Exploratory longitudinal profiling in one patient identified reduced myeloid inflammatory activity and antigen-presentation signatures, attenuated predicted inflammatory intercellular communication, and a more dispersed pattern of T- and B-cell clonal expansion after prolonged treatment.

**Conclusion:**

The clinical cohort findings support the effectiveness and favorable safety profile of Efgartigimod in routine practice. The exploratory single-cell and immune repertoire analyses from one patient provide preliminary insights into potential immunological changes beyond IgG reduction and require validation in larger cohorts.

## 1 Introduction

Myasthenia gravis (MG) is an autoimmune disorder mediated by immunoglobulin G (IgG) autoantibodies targeting the neuromuscular junction (NMJ), leading to impaired neuromuscular transmission through multiple pathogenic mechanisms (1). Approximately 85% of patients harbor antibodies against the acetylcholine receptor (AChR), whereas 6% are positive for muscle-specific kinase (MuSK) antibodies (2). Nearly 10–15% of patients remain seronegative by conventional assays (2, 3); however, this does not necessarily indicate the absence of autoantibodies, as more sensitive approaches such as cell-based assays can detect previously unrecognized antibodies in a subset of cases (4–6). In addition, alternative NMJ-related targets have been identified (7). Although absolute autoantibody titers do not always correlate with disease severity, longitudinal changes in antibody levels are closely associated with disease activity (8, 9). Therefore, reducing circulating pathogenic IgG represents a central therapeutic strategy, particularly in patients with acute exacerbations or myasthenic crisis (10).

Plasma exchange and intravenous immunoglobulin are widely used to rapidly reduce IgG levels; however, both approaches have inherent limitations, including procedural complexity and the risk of adverse events. These limitations highlight the need for more targeted, convenient, and safer strategies for IgG reduction in MG. Efgartigimod, a neonatal Fc receptor (FcRn) blockade, has recently emerged as a novel targeted therapy for anti-AChR antibody-positive generalized MG (gMG) (11). FcRn plays a key role in maintaining IgG homeostasis by rescuing IgG from lysosomal degradation (12, 13). By competitively binding to FcRn, Efgartigimod blocks this recycling pathway and promotes IgG degradation, leading to a rapid and selective reduction in circulating IgG levels without directly suppressing antibody-producing cells (14). Clinical trials and real-world studies have consistently demonstrated that Efgartigimod is safe and well tolerated, with rapid and sustained improvements in disease symptoms and quality of life (15, 16).

The sustained clinical benefits observed by Efgartigimod suggest that its therapeutic effects may extend beyond IgG depletion alone. Emerging evidence suggests that FcRn blockade may influence peripheral immune cell composition and function; however, current findings are not yet fully aligned. Some studies have reported reductions in non-classical monocytes and Th17.1 cells, accompanied by increases in antibody-producing B cells and effector-memory CD4^+^ and CD8^+^T, whereas others have described decreased frequencies of B and T cell subsets (17–19). These discrepancies highlight the complexity of the immunological effects of Efgartigimod and underscore the need for a more comprehensive and high-resolution characterization of immune alterations. Single-cell transcriptomic technologies provide a powerful approach to dissect immune heterogeneity and functional states at the cellular level. Although a recent single-cell study suggested that FcRn blockade may suppress Th17 proliferation and downregulate antigen presentation pathways (20), the longitudinal immunological effects of Efgartigimod, particularly over extended follow-up periods, remain incompletely explored.

In this study, we conducted a real-world clinical investigation to evaluate the efficacy and safety of Efgartigimod in patients with gMG. In addition, we performed an exploratory longitudinal immune profiling study in one patient using paired single-cell RNA sequencing combined with T-cell receptor and B-cell receptor (TCR/BCR) repertoire analysis to investigate potential immunological changes associated with treatment. By integrating clinical outcomes with high-resolution immune profiling, this study aims to provide mechanistic insights into the immunomodulatory effects of FcRn blockade and to facilitate the identification of potential biomarkers for therapeutic response.

## 2 Methods

### 2.1 Participants

Patients with MG who received Efgartigimod at the Second Affiliated Hospital of Harbin Medical University between March 1, 2024 and January 30, 2026 were retrospectively enrolled (Supplementary Table 1). This study consisted of a retrospective clinical cohort study and an exploratory immune profiling substudy; the latter included one patient from the clinical cohort who underwent longitudinal single-cell RNA sequencing and TCR/BCR repertoire analyses after 6 months of treatment. Eligibility criteria were: age ≥18 years, a confirmed diagnosis of gMG, seropositivity for anti–AChR antibodies, and receipt of at least one treatment cycle. Patients were excluded if they were positive for anti-MuSK or anti-low-density lipoprotein receptor-related protein 4 (LRP4) antibodies, were triple seronegative, had comorbidities that could confound efficacy assessment (e.g., other autoimmune diseases, malignancies, or active infections), severe psychiatric or cognitive disorders, or incomplete clinical data or follow-up. Baseline characteristics were collected, including age, sex, age at onset, disease duration, Myasthenia Gravis Foundation of America (MGFA) classification, history of thymectomy, comorbidities, and concomitant medications. Written informed consent was obtained from all participants. The study was approved by the Institutional Review Board of the Second Affiliated Hospital of Harbin Medical University and conducted in accordance with the Declaration of Helsinki.

### 2.2 Treatment and follow-up

Efgartigimod was administered intravenously at 10 mg/kg once weekly for four consecutive weeks. A second cycle was initiated 1 month after completion of the first, with the same regimen. During the subsequent flexible phase, additional cycles were administered at variable intervals according to individualized clinical assessment, considering disease activity, maintenance of clinical benefit, and physician–patient shared decision-making. Concomitant symptomatic and immunosuppressive therapies were recorded throughout follow-up. These treatments were not mandated to remain unchanged; rather, adjustments, including gradual corticosteroid tapering, were made according to clinical response and routine clinical practice. Clinical efficacy was assessed using Myasthenia Gravis Activities of Daily Living (MG-ADL) scores. Changes in concomitant medications were recorded during follow-up.

Clinically meaningful improvement (CMI) was defined as a ≥ 2-point reduction in MG-ADL score from baseline. Minimal symptom expression (MSE) was defined as an MG-ADL score of 0–1.

### 2.3 The single-cell exploratory case

One patient from the retrospective cohort was selected for paired single-cell RNA sequencing and TCR/BCR repertoire analysis at baseline and after 6 months of continuous Efgartigimod treatment.

The patient was a 72-year-old man at enrollment who had developed bilateral ptosis at disease onset at the age of 67 years. After diagnosis, he was treated with pyridostigmine and prednisone, and prednisone was discontinued after 1 year. During the 6 months prior to enrollment, the patient developed chewing fatigue and limb weakness, while remaining without immunosuppressive therapy. At baseline, anti–AChR antibody levels were >20 nmol/L, and titin antibody levels were 27.29 U/mL. Low-frequency repetitive nerve stimulation showed a decremental response in the bilateral orbicularis oculi muscles. Chest computed tomography revealed no thymic abnormalities. Rheumatologic and immunologic screening was otherwise unremarkable, except for mildly elevated anti–double-stranded DNA antibodies (26.24 IU/mL; reference range: 0–10 IU/mL). The baseline serum IgG level was 13.10 g/L.

### 2.4 Sample preparation and sequencing

Peripheral blood (5 mL) was collected, and peripheral blood mononuclear cells (PBMCs) were isolated by Ficoll density-gradient centrifugation. Cells were washed, subjected to red blood cell lysis, and resuspended in phosphate-buffered saline supplemented with 5% fetal bovine serum. Cell count and viability were assessed, and the suspension was adjusted to 600–1,200 viable cells/μL. Single-cell libraries were prepared using the Chromium Next GEM Single Cell 5′ platform (10x Genomics) according to the manufacturer’s instructions. Libraries were sequenced on an Illumina NovaSeq 6,000 platform with a minimum depth of 30,000 reads per cell using a paired-end 150 bp strategy. Raw data were processed using Cell Ranger and aligned to the human reference genome GRCh38 for gene expression quantification.

### 2.5 Bioinformatics analysis

#### 2.5.1 Quality control, dimensionality reduction, cell clustering and annotation

Sequencing data were processed using Seurat (v4.4.0) (21). Cells with <500 detected genes, <1,000 total counts, or >20% mitochondrial gene expression were excluded, and doublets were removed using DoubletFinder (22). Batch effects between pre- and post-treatment samples were corrected using Harmony. Data were normalized, and highly variable genes were identified for downstream analysis. Dimensionality reduction was performed using principal component analysis, followed by clustering and visualization with uniform manifold approximation and projection (UMAP). Cell types were annotated based on canonical marker genes.

#### 2.5.2 Identification of differentially expressed genes

Differentially expressed genes (DEGs) between pre- and post-treatment samples were identified using FindMarkers. Genes with |avg_log2FC| > 0.3 and p_val_adj < 0.05 were considered significant. To reduce the influence of technical artifacts and non-informative signals, mitochondrial genes, ribosomal genes, and erythrocyte-related genes were excluded from downstream analyses.

#### 2.5.3 Functional enrichment analysis

Gene Ontology (GO) enrichment analysis was performed using clusterProfiler (23). Significantly enriched pathways were defined by an adjusted *p* value <0.05. Hallmark gene set activity was quantified at the single-cell level using AUCell.

#### 2.5.4 Cell–cell communication analysis

Cell–cell communication analysis was performed using CellChat (v1.6.1) (24). Normalized gene expression data and annotated cell identities were used as input. Intercellular communication probabilities were inferred based on known ligand–receptor interactions from the CellChatDB.human database, focusing on the “Secreted Signaling” category. Communication networks were constructed at the pathway level, and information flow was calculated to quantify overall signaling strength. Outgoing and incoming signaling patterns were analyzed to characterize cell-type-specific signaling roles. Differences between pre- and post-treatment samples were assessed by comparing interaction strength and number, and results were visualized using heatmaps and bar plots.

#### 2.5.5 Single-cell immune repertoire analysis

Single-cell TCR and BCR repertoire analyses were performed using scRepertoire (25). Clonotypes were defined based on shared CDR3 amino acid sequences and V(D)J gene usage. Clonal expansion, repertoire composition, and CDR3 length were assessed. Differences in clonotype abundance between pre- and post-treatment samples were compared, and clonal homeostasis was evaluated. Clonotype data were integrated with transcriptomic profiles to enable visualization of clonal expansion across cell types.

### 2.6 Statistical analysis

All statistical analyses and data visualization were performed using R software (RStudio version 4.4.2). The normality of continuous variables was assessed using the Shapiro–Wilk test. Normally distributed continuous variables were presented as mean ± standard deviation (SD), whereas non-normally distributed continuous variables were summarized as median (interquartile range, IQR). Categorical variables were expressed as number (percentage). For longitudinally repeated measurements involving three or more time points, the Friedman test was used to assess overall differences across visits. *Post hoc* pairwise comparisons were performed using two-sided Wilcoxon signed-rank tests with Holm correction for multiple comparisons. The same approach was applied to ordinal data. All statistical tests were two-sided, and *p* < 0.05 was considered statistically significant.

## 3 Results

### 3.1 Baseline clinical features

A total of 21 AChR-positive gMG patients were included, all of whom received at least one cycle of Efgartigimod. Twenty-one patients were assessed at V1–V4, 20 at V5–V7, and 19 at V8. Two patients did not complete both treatment cycles. One discontinued Efgartigimod after V4 because of an insufficient clinical response, with MG-ADL scores of 6, 3, 6, and 6 at V1–V4, respectively. The other discontinued treatment after V7 following achievement of MSE and chose not to receive further Efgartigimod treatment. Baseline characteristics are summarized in Table 1. The mean age at disease onset was 60.6 ± 12.1 years. All patients received pyridostigmine prior to Efgartigimod. Immunosuppressive therapy was used in 4 patients, including prednisone (*n* = 1, 4.8%) and tacrolimus (*n* = 3, 14.3%). Thymoma was present in 5 patients (23.8%), of whom 3 had undergone thymectomy. At treatment initiation, MGFA classification was II in 5 patients (23.8%), III in 12 (57.1%), and IV in 4 (19.0%).

**Table 1:** Demographic and clinical characteristics of the patients.

Clinical variables	Mean ± SD or *n* (%)
Onset age (years)	60.6 ± 12.1
Sex (female%)	12 (57.1%)
Disease duration (years)	5.6 ± 2.9
Thymoma (%)	5 (23.8%)
After thymectomy (%)	3 (14.3%)
MGFA class at Efgartigimod initiation	
II	5 (23.8%)
III	12 (57.1%)
IV	4 (19.0%)
V	0 (0.0%)
Previous treatment	
Pyridostigmine	21 (100.0%)
Prednisone	1 (4.8%)
Tacrolimus	3 (14.3%)

### 3.2 The clinical effect of Efgartigimod

MG-ADL outcomes are summarized in Table 2 and Figure 1. MG-ADL scores decreased significantly over the course of Efgartigimod treatment (Figure 1B). The overall difference across visits was significant (Friedman test, *p* < 0.001), and post-hoc analyses showed significantly lower MG-ADL scores at each follow-up visit (V2–V8) compared with baseline (V1) (all Holm-adjusted *p* < 0.001) (Supplementary Table 2). The median MG-ADL score decreased from 7.0 (IQR, 6.0–8.0) at baseline to 3.0 (2.0–4.0) at week 4 (V4), 2.0 (2.0–3.0) at week 9 (V5), and 1.0 (1.0–1.5) at week 12 (V8) (Table 2). Correspondingly, the median relative reduction from baseline was 66.7% (IQR, 50.0–71.4%) at week 4, 71.4% (63.4–77.8%) at week 9, and 85.7% (78.9–87.5%) at week 12. At the end of the first treatment cycle (V4), 18 of 21 patients (85.7%) achieved CMI, and 12 of 21 (57.1%) achieved a ≥ 5-point reduction from baseline. By the end of the second treatment cycle (V8), 18 of 19 patients (94.7%) achieved CMI, and 16 of 19 (84.2%) achieved a ≥ 5-point reduction.

**Table 2:** Clinical efficacy of Efgartigimod in patients with gMG.

Clinical outcome	*N*	Median (IQR)	Holm-adjusted *p* value
Baseline MG-ADL (V1)	21	7.0 (6.0–8.0)	–
MG-ADL (W4, V4)	21	3.0 (2.0–4.0)	<0.001
MG-ADL (W9, V5)	20	2.0 (2.0–3.0)	<0.001
MG-ADL (W12, V8)	19	1.0 (1.0–1.5)	<0.001
Change in MG-ADL (W4, V4), %	21	66.7 (50.0–71.4)	–
Change in MG-ADL (W9, V5), %	20	71.4 (63.4–77.8)	–
Change in MG-ADL (W12, V8), %	19	85.7 (78.9–87.5)	–

“Change in MG-ADL” was defined as the relative reduction from baseline for each patient, calculated as (baseline MG-ADL − follow-up MG-ADL)/baseline MG-ADL × 100%. Data are presented as median (IQR). *p* values were obtained using two-sided Wilcoxon signed-rank tests comparing each follow-up visit with baseline (V1) and were adjusted for multiple comparisons using the Holm method. MG-ADL, Myasthenia Gravis Activities of Daily Living; IQR, interquartile range.

**Figure 1:**
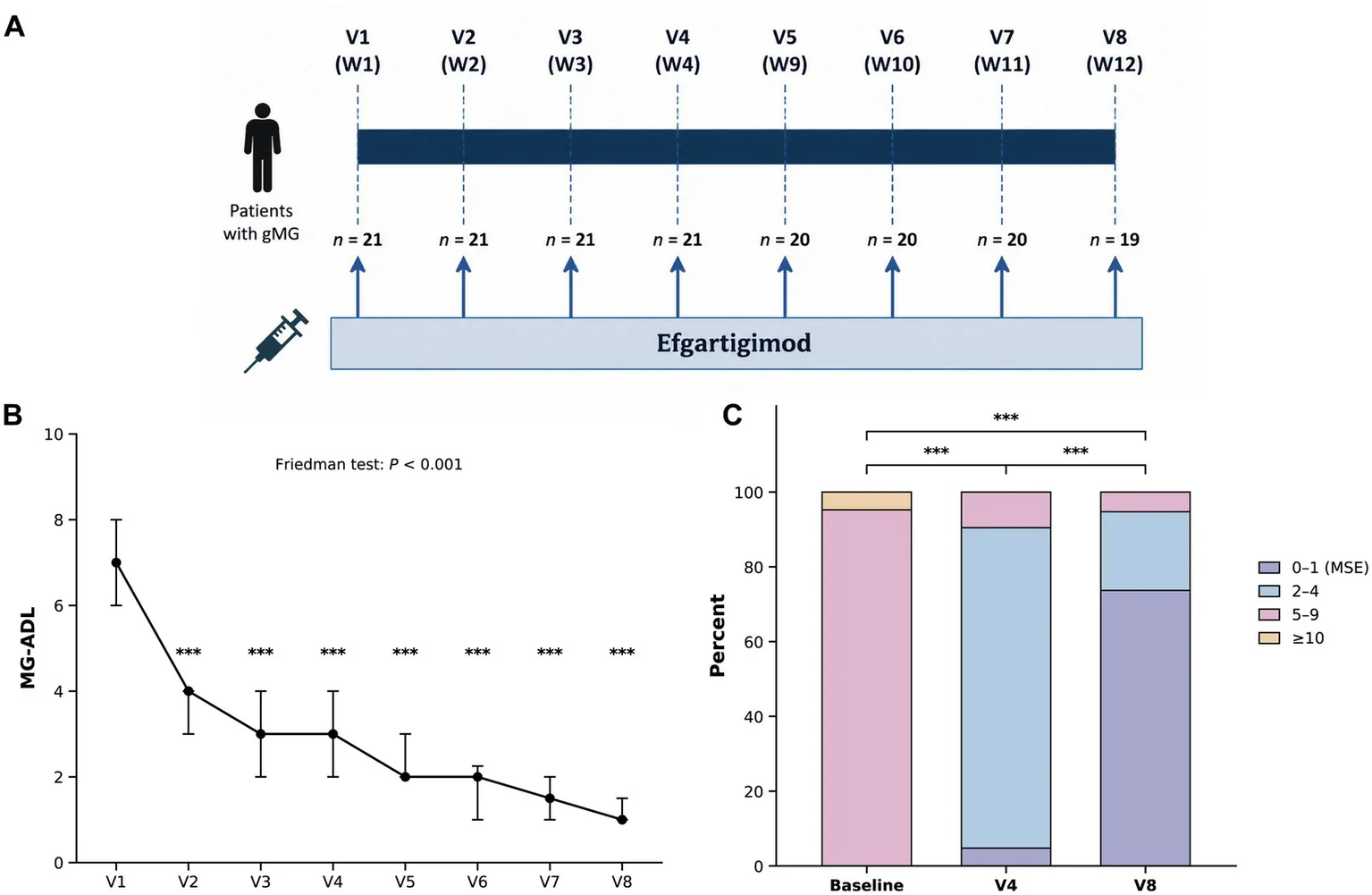
Study design and longitudinal changes in MG-ADL scores during Efgartigimod treatment in patients with gMG. **(A)** Schematic illustration of the treatment and follow-up schedule. Patients received Efgartigimod infusions across two treatment cycles, with clinical assessments performed at scheduled time points (V1–V8 corresponding to weeks 1, 2, 3, 4, 9, 10, 11, and 12). The numbers of patients assessed at each visit were 21 at V1–V4, 20 at V5–V7, and 19 at V8. **(B)** Longitudinal changes in MG-ADL scores over the course of treatment. Data are presented as median (IQR). Overall differences across visits were assessed using the Friedman test in patients with complete observations across V1–V8 (*n* = 19). Post-hoc comparisons between V1 and each follow-up visit were performed using two-sided Wilcoxon signed-rank tests based on available paired observations, with *p* values adjusted for multiple comparisons using the Holm method. Sample sizes at each visit are shown in panel A. *** Holm-adjusted *p* < 0.001 vs. V1. **(C)** Distribution of MG-ADL severity categories at baseline, V4, and V8. MG-ADL scores were categorized as 0–1 (MSE), 2–4, 5–9, and ≥10. Overall differences in ordinal MG-ADL categories across the three time points were assessed using the Friedman test in patients with complete observations (*n* = 19; *p* < 0.001). *Post-hoc* pairwise comparisons were performed using two-sided Wilcoxon signed-rank tests based on available paired observations, with *p* values adjusted for three comparisons using the Holm method. Sample sizes at each visit are shown in panel A. *** Holm-adjusted *p* < 0.001. MG-ADL, Myasthenia Gravis Activities of Daily Living; IQR, interquartile range; MSE, minimal symptom expression.

Consistent with the reduction in MG-ADL scores, the distribution of MG-ADL severity categories shifted toward lower score ranges during treatment (Figure 1C). At baseline, 20 of 21 patients (95.2%) had MG-ADL scores of 5–9 and one (4.8%) had a score ≥10. At week 4 (V4), 18 of 21 patients (85.7%) had scores of 2–4, one (4.8%) had achieved MSE, and two (9.5%) had scores of 5–9. By week 12 (V8), 14 of 19 patients (73.7%) had achieved MSE, four (21.1%) had scores of 2–4, and one (5.3%) had a score of 5–9. The distribution of ordinal MG-ADL categories differed significantly across baseline, V4, and V8 (Friedman test, *p* < 0.001), with all post-hoc pairwise comparisons remaining significant after Holm adjustment (all adjusted *p* < 0.001).

One patient received concomitant prednisone therapy during Efgartigimod treatment. The prednisone dose remained at 20 mg/day from V1 to V5, was reduced to 17.5 mg/day at V6, and was subsequently tapered by 2.5 mg every 2 weeks, reaching 15 mg/day at V8. Thereafter, this patient continued Efgartigimod treatment with pyridostigmine and a reduced prednisone dose, with sustained clinical stability. This individual case illustrates the possibility of corticosteroid reduction during Efgartigimod treatment.

### 3.3 Safety

Efgartigimod was well tolerated, with no serious adverse events. Two treatment-related adverse events were reported: one case of low-grade fever and one case of herpes simplex infection, both of which resolved within 3 days. Treatment was continued after symptom resolution.

### 3.4 Efgartigimod alters peripheral immune cell composition and transcriptional profiles in AChR-positive gMG

To investigate the immunological effects of Efgartigimod in MG, PBMCs from an exploratory longitudinal case were analyzed by single-cell transcriptomics and immune repertoire sequencing at two time points: baseline (prior to treatment) and after treatment. At baseline, the patient had been receiving pyridostigmine for at least 1 year without additional immunosuppressive therapy, with an MG-ADL score of 6. Following baseline sampling, the patient received two standard cycles of Efgartigimod (Figure 2A), followed by maintenance treatment every 2–3 weeks. After a total of 14 infusions over 6 months, peripheral blood samples were collected again. During treatment, the MG-ADL score decreased to 1 after the second cycle and remained stable thereafter (Figure 2B). Serum IgG levels declined during the initial cycles, reached a nadir at V4, showed a transient rebound at V5, and reached the lowest level at V8, without exceeding baseline levels throughout (Figure 2C; Supplementary Table 3).

**Figure 2:**
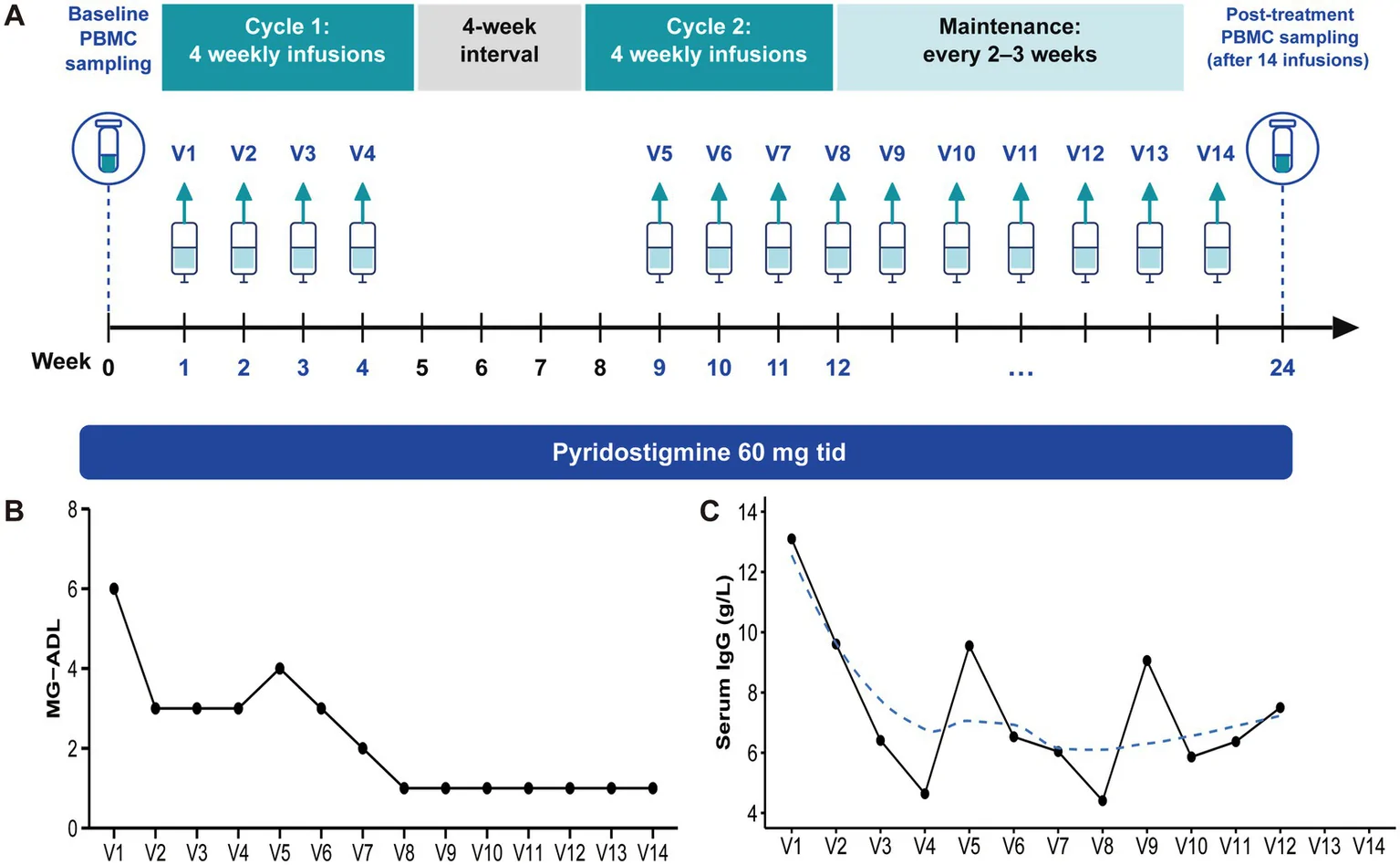
Longitudinal changes in MG-ADL scores and serum IgG levels during treatment in the patient selected for single-cell analysis. **(A)** Overview of PBMC sampling and Efgartigimod administration. **(B)** MG-ADL scores over time, showing rapid and sustained clinical improvement. **(C)** Serum IgG levels over time. The dashed line indicates the fitted trend.

After quality control, 25,272 PBMCs were retained for analysis. Based on canonical markers, cells were classified into six major types: CD4^+^ T cells, CD8^+^ T cells, NK cells, B cells, monocytes, and dendritic cells (DCs), and further resolved into 13 subtypes (Figures 3A–C). UMAP revealed distinct shifts in cellular distribution after treatment, with reduced myeloid populations and increased NK cell density (Figure 3A). Accordingly, cell proportion analysis showed a decrease in monocytes and an expansion of NK cells, while effector-memory CD4^+^ and CD8^+^ T-cell populations showed a slight increasing trend (Figure 3D). Supporting our observations, a recent flow cytometric study reported a similar trend after Efgartigimod treatment, characterized by reduced monocyte populations and increased effector-memory CD4^+^ and CD8^+^ T-cell populations (17). Transcriptional changes were most evident in monocytes/DCs (Figure 3E).

**Figure 3:**
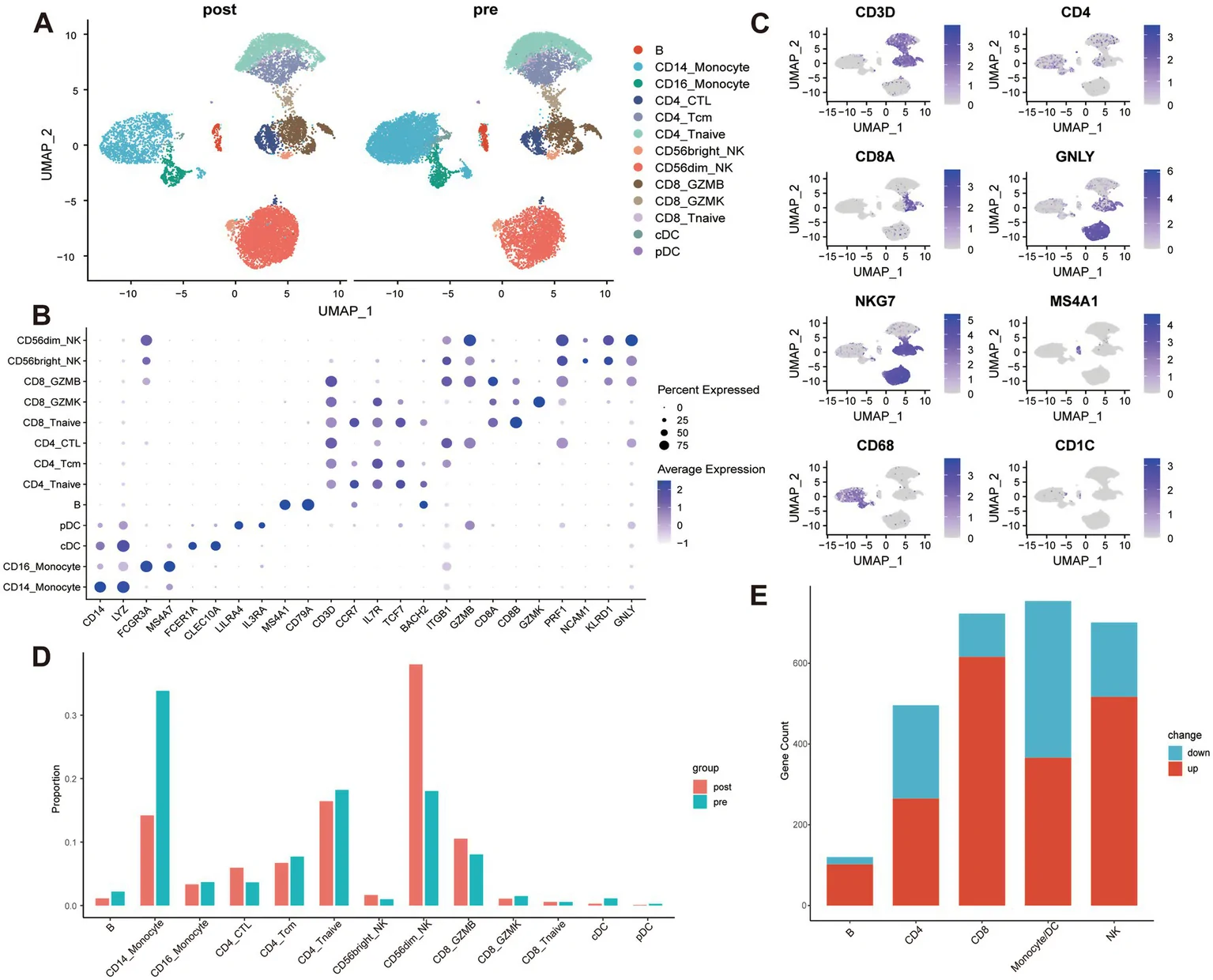
Single-cell atlas of peripheral immune landscape before and after Efgartigimod treatment in anti-AChR antibody positive gMG. **(A)** UMAP visualization of PBMCs before and after treatment. **(B)** Canonical marker gene expression across annotated cell subtypes. **(C)** Feature plots of representative marker genes. **(D)** Cellular composition by group. **(E)** Differentially expressed genes across major cell types.

### 3.5 Efgartigimod attenuates inflammatory and antigen-presenting pathways in myeloid cells while modulating cytotoxicity of NK cells

DEGs in monocytes/DCs between post- and pre-treatment samples are shown in Figure 4A. GO enrichment of downregulated genes revealed significant suppression of immune-related pathways (Figure 4B; Supplementary Table 4). In particular, inflammatory signaling pathways, including NF-κB, IL-6, TNF, IL-12, IL-23, and type I/II interferon responses, were broadly reduced. Previous studies have reported treatment-associated changes in inflammatory signals, suggesting a reduction in IL-6 levels following treatment (18, 26). Pathways involved in antigen processing and presentation, as well as immune cell migration (e.g., chemotaxis and leukocyte migration), phagocytosis, and Fc receptor–mediated signaling, were also decreased. These findings indicate a coordinated attenuation of inflammatory activity and antigen-presenting capacity in monocytes/DCs following Efgartigimod treatment. Consistent with previous reports20, FcRn blockade may dampen myeloid activation and reduce chemotactic signaling and antigen presentation. AUCell analysis further confirmed a global reduction in inflammatory pathway activity at the single-cell level (Figures 4D–F). Hallmark pathways, including inflammatory response, IL-6–JAK–STAT3, and TNFα–NF-κB signaling, were enriched in monocytes/DCs before treatment but markedly decreased afterward.

**Figure 4:**
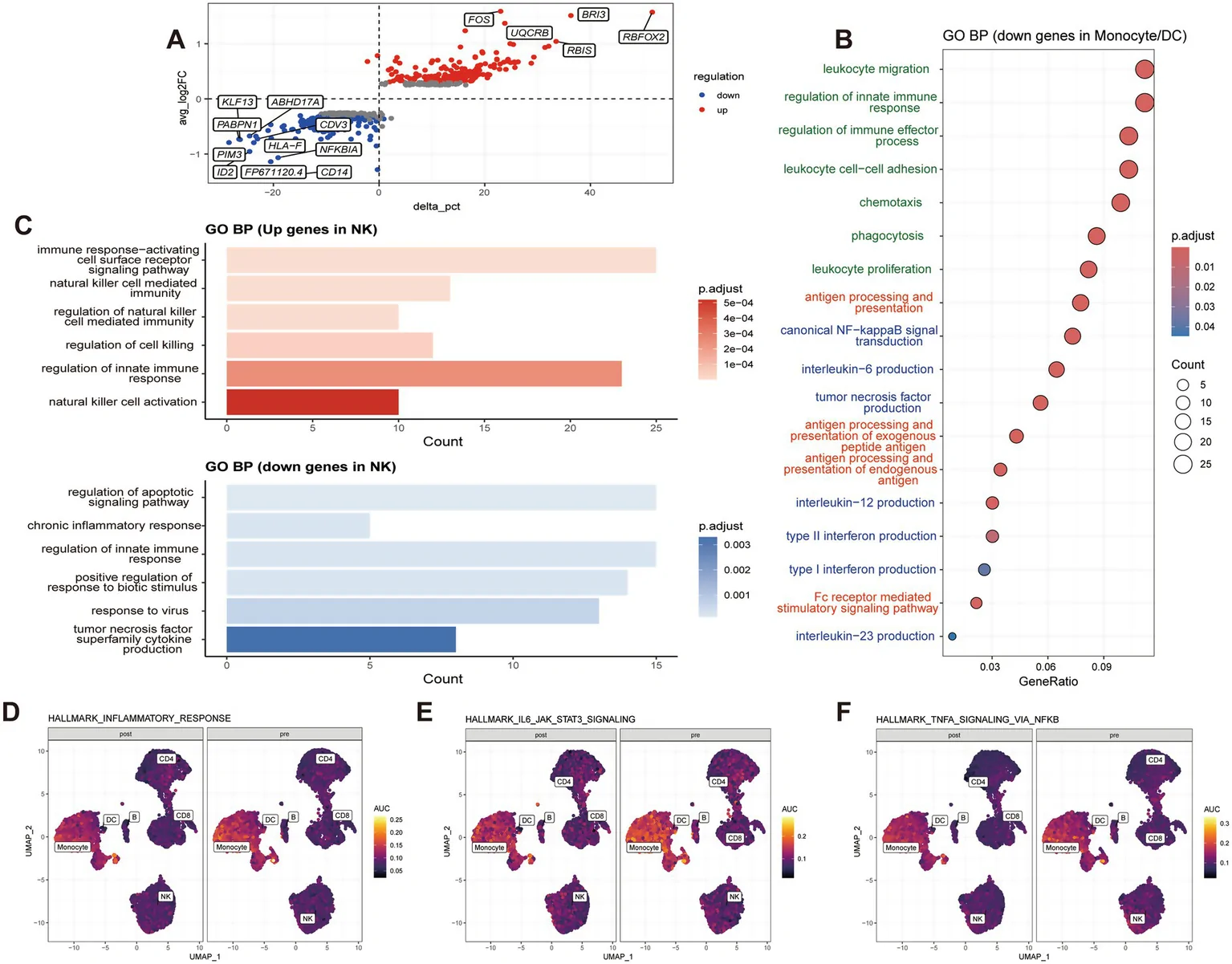
Differential expression and functional enrichment analyses of myeloid and NK cells before and after Efgartigimod treatment. **(A)** Volcano plot showing DEGs in monocytes/DCs. Upregulated and downregulated genes are highlighted in red and blue, respectively. Selected representative genes are labeled. **(B)** Gene Ontology (GO) biological process enrichment analysis of downregulated genes in Monocytes/DCs. **(C)** GO biological process enrichment analysis of DEGs in NK cells. **(D–F)** AUCell-based visualization of pathway activity scores for **(D)** inflammatory response, **(E)** IL-6–JAK–STAT3 signaling, and **(F)** TNFα–NF-κB signaling before and after Efgartigimod treatment. Color intensity represents AUC scores, and cell-type annotations are indicated on the UMAP plots.

GO biological process enrichment of DEGs in NK cells showed that upregulated genes were enriched in immune activation–related pathways, including NK cell–mediated immunity, regulation of cell killing, and innate immune responses, suggesting enhanced cytotoxicity (Supplementary Table 5). In contrast, downregulated genes were enriched in pathways related to apoptotic signaling, chronic inflammation, and TNF superfamily cytokine production (Supplementary Table 6). Overall, these findings suggest a functional shift in NK cells toward increased cytotoxicity with reduced inflammatory activity (Figure 4C).

### 3.6 Efgartigimod alters intercellular communication, characterized by reduced inflammatory and enhanced immunosuppressive signaling

Cell–cell communication was analyzed to characterize ligand–receptor interactions among major immune cell populations before and after treatment. Monocytes and DCs exhibited the highest levels of outgoing and incoming signaling, indicating central roles in the communication network (Figure 5A). Following treatment, both the strength and number of intercellular interactions were altered. Interaction strength analysis showed a global reduction in monocyte-associated signaling, either as senders or receivers, particularly in interactions with CD4^+^ T cells and among monocytes (Figure 5C). In contrast, NK cells exhibited increased incoming signaling. Interaction number analysis revealed decreased DC-associated interactions and increased interactions between NK cells and T or B cells (Figure 5D). These findings suggest reduced myeloid-dominated communication and relatively enhanced NK cell–associated interactions after treatment.

**Figure 5:**
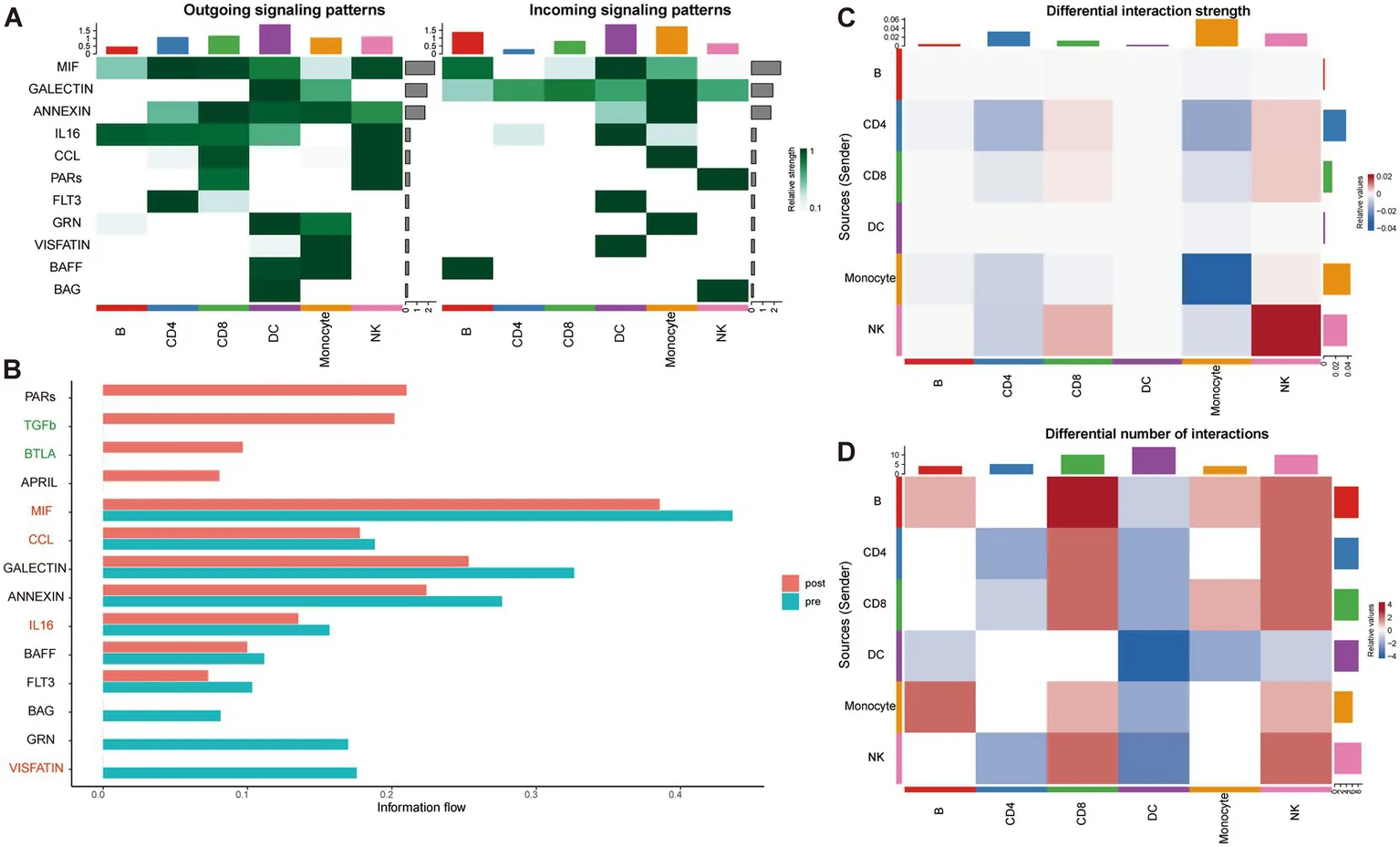
CellChat analysis of intercellular communication dynamics among major immune cell populations before and after treatment. **(A)** Outgoing and incoming signaling patterns showing the distribution of signal-sending and signal-receiving roles across different immune cell types. **(B)** Comparison of pathway-specific information flow between pre- and post-treatment conditions. **(C)** Differential interaction strength between immune cell populations. Each cell pair represents changes in signaling strength from sender (rows) to receiver (columns). **(D)** Differential number of interactions between immune cell populations.

Quantitative analysis of pathway-specific information flow (Figure 5B) revealed significant changes in multiple immune-related signaling pathways. Compared with pre-treatment, inflammatory and chemotactic pathways, including MIF, CCL, IL-6, and VISFATIN, showed reduced information flow after treatment. In contrast, TGF-β and BTLA signaling were increased. TGF-β is a key immunoregulatory cytokine involved in maintaining immune homeostasis and tolerance (27, 28), whereas BTLA is an inhibitory receptor that suppresses lymphocyte activation and proliferation and has been implicated in immune regulation (29, 30). These results indicate a reorganization of the intercellular communication network, with reduced inflammatory and chemotactic signaling alongside enhanced immunosuppressive pathways.

### 3.7 Efgartigimod weakens antigen-driven clonal dominance

TCR sequencing analysis characterized the distribution patterns of CDR3 lengths. For both TRA and TRB chains, the number of clonotypes at identical CDR3 lengths was reduced in post-treatment samples compared with pre-treatment samples (Figure 6A), and the proportion of unique clonotypes also decreased (Figure 6B). Clonal homeostasis analysis indicated increased clonal expansion, reflected by a higher proportion of large clones (0.01 < X ≤ 0.1) and fewer small clones (1e−04 < X ≤ 0.001) (Figure 6C). In contrast, clonal space analysis showed a decrease in extremely expanded clones (clone size >2,500) (Figure 6D). Together, these findings suggest a shift from oligoclonal dominance toward a more distributed clonal expansion pattern. Integration with transcriptomic data showed that clonal expansion was predominantly enriched in effector T cell subsets, including CD8_GZMB, CD4_CTL, and CD8_GZMK cells (Figures 6E,F).

**Figure 6:**
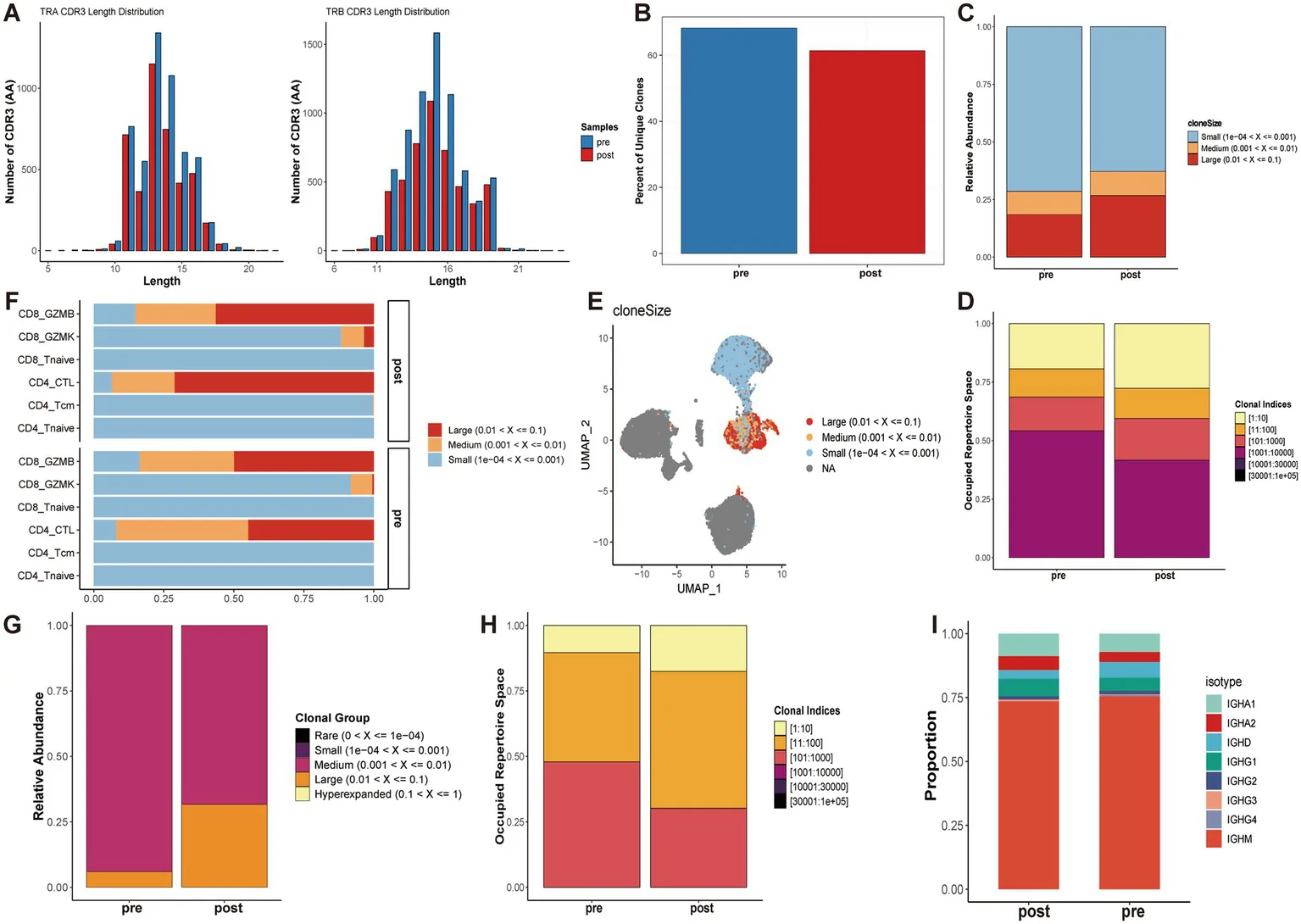
Comprehensive analysis of TCR and BCR repertoire dynamics before and after treatment. **(A)** Distribution of CDR3 lengths for TRA and TRB chains. **(B)** Proportion of unique TCR clonotypes in pre- and post-treatment samples. **(C)** TCR clonal homeostasis analysis showing the relative abundance of small, medium, and large clones. **(D)** TCR clonal space analysis illustrating the proportion of repertoire occupied by clones of different sizes. **(E)** UMAP visualization of T cells colored by clone size, showing the spatial distribution of expanded clonotypes. **(F)** Distribution of clone sizes across T cell subsets, indicating enrichment of clonal expansion in effector T cell populations. **(G)** BCR clonal homeostasis analysis showing the relative abundance of medium and large clones. **(H)** BCR clonal space distribution across different clone size categories. **(I)** BCR isotype distribution in pre- and post-treatment samples.

Exploratory B-cell subclustering identified six transcriptionally distinct B-cell states and revealed descriptive changes in subset composition before and after Efgartigimod treatment (Supplementary Figure 1). Further BCR sequencing analysis showed a corresponding shift in clonal architecture, from oligoclonal dominance to polyclonal expansion (Figures 6G,H). The BCR repertoire was predominantly composed of IGHM (~70–75%), and isotype distribution remained largely stable after treatment, with only a slight increase in IgG and IgA and no clear evidence of class-switch recombination (Figure 6I).

Taken together, these findings indicate a reshaping of the clonal architecture toward a more balanced and evenly distributed immune repertoire following Efgartigimod treatment.

## 4 Discussion

Targeted therapies for MG have evolved from B-cell depletion (31, 32) to complement inhibition (33, 34) and, more recently, FcRn blockade (11), reflecting a shift toward precise modulation of pathogenic IgG-mediated mechanisms. As a first-in-class FcRn inhibitor, Efgartigimod has been supported by growing real-world evidence in AChR-positive gMG (35–38). Consistent with these observations, our study showed that after two treatment cycles, 94.7% of patients achieved CMI and 73.7% reached MSE, with only mild adverse events observed, further supporting its favorable efficacy–safety profile in real-world settings. Efgartigimod has shown clear advantages in achieving MSE (39), particularly in rapidly controlling symptoms during myasthenic crisis (40), and enables steroid-sparing effects (41). Beyond its approved indications, Efgartigimod has also demonstrated efficacy in MuSK-positive MG and ocular MG (42, 43) as well as in other IgG-mediated autoimmune diseases, including immune thrombocytopenia, autoimmune encephalitis, neuromyelitis optica spectrum disorder, and chronic inflammatory demyelinating polyradiculoneuropathy (44–47), highlighting its broad translational potential.

With the expanding clinical use of Efgartigimod, increasing attention has focused on its immunomodulatory effects beyond IgG reduction. Dynamic changes in inflammatory mediators, including decreased FGF-19 and IL-41 levels, have been reported during Efgartigimod treatment (26, 48). At the cellular level, Efgartigimod has been shown to modulate DCs, downregulating MHC class II and costimulatory molecules, thereby potentially impairing antigen presentation (49). In our study, we observed treatment-associated changes in the peripheral immune landscape, including altered proportions of myeloid-cell populations and transcriptional shifts within these populations. Functional enrichment analyses further showed downregulation of inflammatory and antigen presentation pathways in myeloid cells, supporting a suppressive effect on innate immune activation, which were directionally consistent with previous reports (20). Mechanistically, FcRn not only regulates IgG recycling but also mediates antigen–IgG immune complex processing and presentation (50, 51), which is associated with proinflammatory responses (52). Given this, it is not surprising that FcRn blockade could attenuate immune complex–driven inflammation, including reduced production of TNF-α and IL-6, as has been demonstrated (53). Our data also demonstrated decreased activity of key inflammatory pathways in myeloid cells, including IL-6, IL-12, IL-23, TNF, and interferon signaling, which are central to MG pathogenesis (54).

Given that monocytes and DCs are major sources of proinflammatory cytokines and chemokines, their functional suppression would be expected to attenuate cell–cell signaling. Our data showed a global reduction in intercellular communication, with monocytes and DCs—serving as central hubs—exhibiting decreased signaling strength and interaction number after treatment. Similar reductions in interaction strength among myeloid cells and CD4^+^ T-cell subsets have been reported following FcRn blockade (20). Within the adaptive immune compartment, we observed a shift in TCR and BCR repertoire architecture from oligoclonal dominance toward more distributed polyclonal expansion. While Hui-Ning Li et al. similarly reported contraction of large clones, they also observed increased IGHG1^+^ B cells and immunoglobulin transcription, interpreted as a compensatory response following antibody depletion (20). Comparable rebound phenomena have been described after plasma exchange (55) but were not evident in our cohort, possibly reflecting differences in sampling timepoints, as our samples were obtained after prolonged treatment (~6 months), when transient rebound effects may have subsided. However, owing to the single-cohort design, inter-individual variability cannot be excluded. We did not perform antigen-specific analyses and therefore cannot directly assess changes in AChR-specific B cells. Nevertheless, previous studies have shown that long-term FcRn blockade can reduce antigen-specific B cells in other autoimmune diseases (56). Whether such rebound phenomena are universal and their temporal dynamics warrant further investigation in larger longitudinal studies.

Across multiple immune dimensions, including immune cell composition, transcriptional programs, intercellular communication, and clonal architecture, our exploratory analyses identified coordinated treatment-associated changes. These observations generate a hypothesis that FcRn blockade may be associated with broader modulation of both innate and adaptive immune responses. This hypothesis is biologically plausible and consistent with the known mechanism of Efgartigimod-mediated IgG reduction, as well as previous studies using complementary approaches that have reported treatment-associated alterations in antigen-presentation pathways, inflammatory signaling, myeloid and lymphocyte states, and immune-repertoire characteristics (17, 18, 20, 26, 57).

Several limitations should be acknowledged. The clinical cohort was relatively small and retrospective, and the single-cell immune analyses were based on longitudinally paired samples from only one patient without biological replication. Therefore, patient-specific temporal variation, technical variability, and potential batch effects cannot be excluded. In addition, flow-cytometric validation of immune-cell subset changes and targeted molecular validation of candidate inflammatory genes were not available in an expanded cohort, limiting the generalizability of the single-cell findings. Although the paired within-patient design reduces inter-individual variability and minimizes confounding from concomitant immunotherapies, the single-cell results remain exploratory and require validation in larger prospective cohorts.

In conclusion, our real-world clinical data support the effectiveness and favorable safety profile of Efgartigimod in patients with gMG. The exploratory longitudinal single-cell analysis provides preliminary observations suggesting treatment-associated changes in myeloid inflammatory activity, antigen-presentation programs, intercellular immune communication, and adaptive immune-cell clonality. These findings provide potential mechanistic context for FcRn-targeted therapy but should not be interpreted as establishing causal immunological effects. Further prospective studies are warranted to validate these observations and clarify their biological implications.

## 5 Conclusion

Our retrospective clinical cohort provides additional real-world evidence supporting the clinical effectiveness and safety of Efgartigimod in patients with gMG. Separately, exploratory longitudinal single-cell analysis in one patient identified potential immunological changes associated with prolonged treatment, including reduced myeloid activation and inflammatory signaling, decreased antigen-presentation activity, and attenuation of antigen-related immune responses. These observations were broadly consistent with the known immunological effects of Efgartigimod and provide hypothesis-generating insights into its potential longer-term immune modulation. Further studies in larger longitudinal cohorts are required to validate these findings and determine their relationship with sustained clinical benefit.

## Data Availability

The original contributions presented in the study are included in the article and Supplementary Material. Further inquiries can be directed to the corresponding authors.
