# Supplementary material for: Real-world clinical effectiveness and exploratory longitudinal immunological changes of Efgartigimod in myasthenia gravis: a retrospective study and a single-cell analysis in one patient
Source: Front Neurol. 2026 Oct 19;17:1980412. doi: 10.3389/fneur.2026.1980412 (PMC13724452; doi:10.3389/fneur.2026.1980412)
Supplement: Supplementary file 1 [file Data_Sheet_1.zip › Supplementary materials/Supplementary Figure.docx]

Supplementary Figure 1. Exploratory characterization of B-cell subsets before and after efgartigimod treatment.

**(A)** UMAP visualization of B-cell clusters identified by secondary clustering. **(B)** UMAP visualization of the annotated B-cell subsets, including naïve B cells, CD83⁺ activated naïve B cells, memory B cells, activated memory B cells, unswitched memory B cells, and T-bet⁺ atypical-like B cells. **(C)** Dot plot showing the expression of representative marker genes used for B-cell subset annotation; dot size represents the percentage of cells expressing each gene and color indicates average expression. **(D)** Relative proportions of the annotated B-cell subsets before and after efgartigimod treatment. Given that the single-cell analysis was derived from one patient, the observed changes in B-cell subset composition are presented descriptively and should be interpreted as exploratory.
