# Supplementary material for: Real-world clinical effectiveness and exploratory longitudinal immunological changes of Efgartigimod in myasthenia gravis: a retrospective study and a single-cell analysis in one patient
Source: Front Neurol. 2026 Oct 19;17:1980412. doi: 10.3389/fneur.2026.1980412 (PMC13724452; doi:10.3389/fneur.2026.1980412)
Supplement: Supplementary file 1 [file Data_Sheet_1.zip › Supplementary materials/Supplementary Table 2.docx]

**Longitudinal changes in MG-ADL scores during efgartigimod treatment**

| Visit | n | MG-ADL, median (IQR) | Median change from baseline (95% CI) | Wilcoxon Signed-Rank Test | |
| --- | --- | --- | --- | --- | --- |
|  |  |  |  | Z | Holm-adjusted P value |
| V1 | 21 | 7.0 (6.0–8.0) | - | - | - |
| V2 | 21 | 4.0 (3.0–4.0) | 3.0 (3.0–3.0) | −4.06 | <0.001 |
| V3 | 21 | 3.0 (2.0–4.0) | 4.0 (4.0–5.0) | −3.95 | <0.001 |
| V4 | 21 | 3.0 (2.0–4.0) | 5.0 (4.0–5.0) | −3.94 | <0.001 |
| V5 | 20 | 2.0 (2.0–3.0) | 5.0 (4.5–6.0) | −3.95 | <0.001 |
| V6 | 20 | 2.0 (1.0–2.25) | 5.0 (5.0–6.5) | −3.94 | <0.001 |
| V7 | 20 | 1.5 (1.0–2.0) | 6.0 (5.0–6.5) | −3.93 | <0.001 |
| V8 | 19 | 1.0 (1.0–1.5) | 6.0 (5.0–7.0) | −3.84 | <0.001 |

Data are presented as median (interquartile range [IQR]). Overall differences in MG-ADL scores across V1–V8 were assessed using the Friedman test in patients with complete observations across all visits (n = 19; χ² (7) = 111.86, P < 0.001). Post-hoc comparisons between baseline (V1) and each follow-up visit (V2–V8) were performed using two-sided Wilcoxon signed-rank tests based on available paired observations, with P values adjusted for seven comparisons using the Holm method. Median changes were calculated as the baseline score minus the corresponding follow-up score; positive values therefore indicate reductions in MG-ADL scores from baseline. CI, confidence interval; IQR, interquartile range; MG-ADL, Myasthenia Gravis Activities of Daily Living.
